# Retinal ganglion cell layer thickness and volume measured by OCT changes with age, sex, and axial length in a healthy population

**DOI:** 10.1186/s12886-022-02488-7

**Published:** 2022-06-24

**Authors:** Abbas Al-Hawasi, Neil Lagali

**Affiliations:** Division of Ophthalmology, Department of Biomedical and Clinical Sciences, Faculty of Medicine, Linköping University, 581 83 Linköping, Sweden

**Keywords:** Ganglion cell layer (GCL), Retinal ganglion cell layer (RGCL), Ganglion cell layer thickness (GCLT), Optical coherence tomography (OCT), Ganglion cell volume (GCV)

## Abstract

**Background:**

The ganglion cell layer (GCL) measurements with Optical Coherence Tomography (OCT) are important for both ophthalmologists and neurologists because of their association with many ophthalmic and neurological diseases. Different factors can affect these measurements, such as brain pathologies, ocular axial length (AL) as well as age and sex. Studies conducted to measure the GCL have overlooked many of these factors. The purpose of this study is to examine the effect of age, sex, and AL on normal retinal GCL thickness and volume in a healthy population without any neurological diseases.

**Methods:**

A prospective cross-sectional study was designed to measure GCL thickness and total volume with OCT with automated segmentation and manual correction where needed. Visual acuity, AL, and autorefraction were also measured. A mixed linear model was used to determine the association of the effect of the various parameters on the GCL thickness and volume.

**Results:**

One hundred and sixteen eyes of 60 subjects (12–76 years of age, 55% female) were examined of which 77% had 0 ± 2 D of spherical equivalent, and mean axial length was 23.86 mm. About 25% of the OCT-automated GCL measurements required manual correction. GCL thickness did not differ in similar anatomic regions in right and left eyes (*P* > 0.05). GCL volume was greater in males relative to females after adjustment for age and axial length (1.13 ± 0.07 mm^3^ for males vs 1.09 ± 0.09 mm^3^ for females; *P* = 0.031). GCL thickness differed between males and females in the inner retinal ring (*P* = 0.025) but not in the outer ring (*P* = 0.66). GCL volume declined with age (*P* = 0.031) but not after adjustment for sex and axial length (*P* = 0.138). GCL volume declined with longer axial length after adjustment for age and sex (*P* = 0.048).

**Conclusion:**

Age, sex and axial length should be taken into consideration when measuring the GCL thickness and volume with OCT. Automated OCT segmentation should be reviewed for manual adjustments.

## Background

The ganglion cell is the first order neuron in the visual pathway, with the cell body located in the retinal GCL while the retinal nerve fiber layer (RNFL) represents the axon of these neurons that leaves the eye and enters the brain through the optic nerve, where it synapses at the lateral geniculate body.

This anatomical separation makes it possible to study the GCL by direct imaging using OCT and consider it as a window into different physiological and pathological processes in the brain [1–6]. The measurement of the RNFL has been thoroughly investigated in different diseases such as glaucoma [7], idiopathic intracranial hypertension (IIH) [8] and optic neuritis [9]; on the other hand, the GCL has also been measured and investigated as the retinal ganglion cell complex (GCC), which includes both the ganglion cell layer and the inner plexiform layer (IPL) [10–12].

With modern OCT devices it is possible to measure the GCL alone and not as a complex with the IPL. Some studies show changes in the GCL with age and sex [13, 14] without considering the effect of axial length, while other studies report GCL thickness changes with axial length [15, 16]. To date, however, no data is available on how the GCL changes taking all three factors into consideration. This relationship, however, is important to investigate as the potential clinical applications of GCL thickness and volume measurement continues to increase in ophthalmological as well as in neurological diseases. Correction for the effects of potential confounders on GCL parameters could be necessary in such investigations. The aim of this study was therefore to examine the GCL by OCT, and determine the variation in its thickness with age, axial length, and sex in a healthy population.

## Method and materials

### Subjects and recruitment

A prospective cross-sectional cohort study was conducted at the Department of Ophthalmology, Linköping University Hospital after obtaining approval from the Linköping Regional Ethics Committee (Approval no. 2015/151–31). Participants were recruited from a population of visitors to the ophthalmology department and their relatives. All participants and/or their parents or their legal guardian(s) gave informed written consent prior to participation. All methods were carried out in accordance with relevant guidelines and regulations. Participants fulfilling any of the following criteria were excluded from the study: any ocular diseases other than mild cataract, history of ocular trauma, diabetes mellitus, any history of cardiovascular disease, carotid artery disease or cerebrovascular accident (CVA), history of radiotherapy to the face and neck, history of chemotherapy, any neurological disease or family history of such disease (eg. multiple sclerosis), or previous treatment with Ethambutol. Any abnormalities or diseases found after consenting were managed according to standard clinical guidelines and the subject was excluded from the study. Recruitment of study subjects occurred during the period October 2015—January 2019.

### Ophthalmic measurements

The visual acuity, axial length, autorefraction/spectacles power, and OCT examination was performed by an expert ophthalmic nurse or ophthalmologist. Visual acuity was measured using the Snellen chart at five meters, and results were noted in decimals. Autorefraction and keratometry were measured using the Topcon® TRK-2P autorefractor-keratometer (Topcon Corp., Japan). The axial length was measured using the Carl Zeiss® IOL master 700 (Carl Zeiss, Meditek, Germany).

The Heidelberg SPECTRALIS OCT system (Heidelberg Engineering, Germany) was used to examine and measure the retinal layers. The keratometry measurement was entered into the OCT examination protocol, and refractive error was included from the most recent spherical equivalent reading prior to OCT examination.

**Fig. 1:**
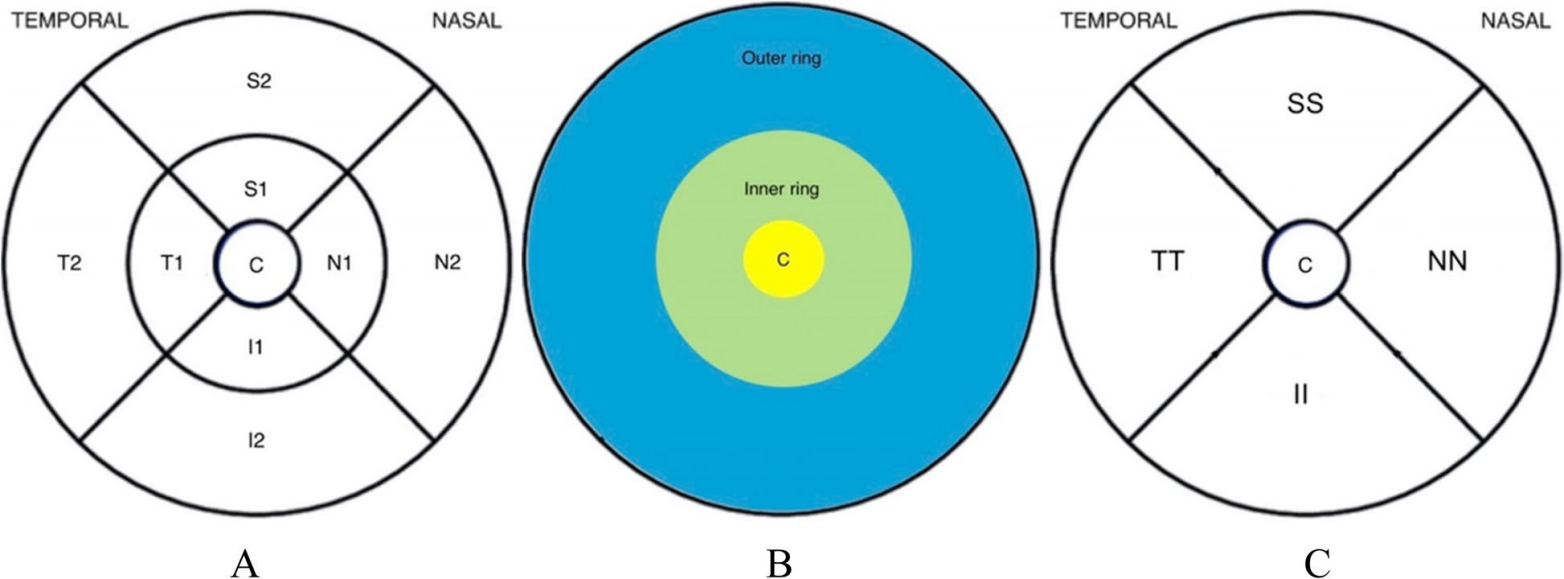
Division of retinal measurements into different anatomic sectors: **A** the 6 mm ETDRS quadrants sectored by the OCT device. S (superior), T (temporal), N (nasal) and I (inferior), 1 for inner sector and 2 for outer sector. **B** Dividing the measured area into central 1 mm ring- C, inner ring 3 mm diameter and outer ring 6 mm., **C** Dividing the inner and outer ring into SS (mean superior of S1 and S2 in figure-A) TT (mean temporal, T1 and T2 in figure-A) NN (mean nasal, N1 and N2 in figure-A) II (mean inferior, I1 and I2 in figure-A)

The Retina dens 30 × 20 degrees program centered on the macula was used. The glaucoma RNFL protocol centered on the optic nerve was examined to identify possible cases of glaucoma. By using the built-in software, the segmentation of different retinal layers was made, and any automated errors in measuring the GCL layer were corrected manually by an experienced clinician. Measurements of different anatomic sectors of the retina were made according to the scheme illustrated in Fig. 1. Optic disc morphology evaluated by fundoscopy through a slit lamp examination or by photography in association with the OCT examination.

Subjects with suspected thinning in the optic nerve head RNFL and/or suspicious appearance of the optic nerve head (in accordance with the European Glaucoma Society Terminology and Guidelines for Glaucoma 3^rd^ edition, 2008) were examined by fundoscopy, IOP and Humphrey visual field 24–2 SITA-fast to exclude glaucoma.

### Statistical analysis

All data was inputted into an Excel file (version 16.59, Microsoft office 365) on a PC, after which data was de-identified and transformed into an SPSS compatible format. For each anatomic retinal sector, a paired t-test was used to compare similar sectors in both eyes. A mixed linear regression model was used for paired-eye analysis and to adjust for axial length, sex and age. Pearson correlation was used to assess volume changes with age for male (normal distribution) while Spearman correlation was used for female subpopulations (non-normal distribution). All statistics were performed using IBM SPSS Statistics, Version 27.0. Armonk, NY: IBM Corp. A two-sided critical *P*-value of < 0.05 indicated significance.

## Results

Data were obtained from 116 eyes of 60 subjects with a mean age of 40.2 years (range 12–76 years). Four eyes were excluded because of 1 eye with epiretinal membrane, 1 eye with previous trauma and 2 eyes with unreliable OCT measurements which could not be manually corrected. 77% of eyes had a spherical equivalent (SE) refraction of 0 ± 2 diopters (D), while the mean axial length for the study population was 23.86 mm (range 22.91–26.82 mm). The demographic characteristics of the study participants are summarized in Table 1.

**Table 1:** The demographic characteristics of the study participants

	Female		Male		All subjects		
Parameter	Mean ± SD	Range	Mean ± SD	Range	Mean ± SD	Range	*P* value
Age (years)	42 ± 17.8	15.6–76.4	38.0 ± 18.1	12–69.9	40.2 ± 18	12–76.4	0.942
Visual acuity (Decimal)	1.1 ± 0.2	0.7–2.0	1.1 ± 0.2	0.6–2.0	1.1 ± 0.2	0.6–2.0	0.547
Axial length (mm)	23.55 ± 0.79	21.98–25.14	24.26 ± 0.96	22.91–26.82	23.86 ± 0.94	21.98–26.82	0.322
		Female	Male	All subjects			
Number of eyes	Right	32	24	56			
	Left	33	27	60			
Spherical equivalent (number of eyes)	0 ± 2 diopters	58	33	91			
	+ 2—+ 4	4	4	8			
	-2—-4	3	11	14			
	-3—-6	0	3	3			

About 25% of the GCL measurements taken by the OCT needed manual correction at some point**.** The GCL thickness across various retinal regions and sectors for both men and women are shown in Table 2. The thickest sectors in both male and female subpopulations separately were S1, I1, N1 followed by T1 respectively, while the thinnest sector apart from C was the I2. There was no significant difference in GCL thickness within a given sector between eyes of the same subject (*P* > 0.05 for all sectors).

**Table 2:** GCL parameter values stratified by sex and by retinal sector. * *P* value < 0.05

		Female		Male		All subjects		*P* value
	Anatomic location	Mean ± SD	Range	Mean ± SD	Range	Mean ± SD	Range	
GCL thickness (µm)	GCL C	14.5 ± 3.2	9–25	16.9 ± 3.9	9–25	15.6 ± 3.7	9–25	0.101
	GCL T1	48.7 ± 4.8	36–60	50.4 ± 3.9	42–60	49.5 ± 4.5	36–60	0.387
	GCL S1	53.3 ± 4.2	44–64	55 ± 4	46–62	54 ± 4.2	44–64	0.858
	GCL N1	52 ± 5	42–63	53.7 ± 4.3	45–63	52.7 ± 4.8	42–63	0.314
	GCL I1	52.8 ± 5	35–64	54.8 ± 4.4	44–64	53.7 ± 4.8	35–64	0.822
	GCL T2	36.7 ± 4.1	29–47	38.3 ± 3.2	31–44	37.4 ± 3.8	29–47	0.052
	GCL S2	35 ± 3.2	29–43	35.8 ± 2.6	30–41	35.4 ± 3	29–43	0.135
	GCL N2	38.4 ± 3.7	30–47	38.6 ± 3.2	33–44	38.5 ± 3.5	30–47	0.186
	GCL I2	33.4 ± 3.7	26–42	33.9 ± 2.4	29–39	33.6 ± 3.2	26–42	< 0.001*
	Inner ring	51.7 ± 4.4	42–63	53.4 ± 3.9	45–62	52.5 ± 4.3	42–63	0.521
	Outer ring	35.9 ± 3.4	29.7–43.7	36.7 ± 2.5	32–41.3	36.2 ± 3	29.8–43.8	0.014*
	TT	42.7 ± 4.1	33.5–53.0	44.4 ± 3.2	38.0–52.0	43.4 ± 3.8	33.5–53.0	0.128
	NN	45.2 ± 3.7	39.0–55.0	46.1 ± 3.2	39.5–53.0	45.6 ± 3.5	39.0–55.0	0.254
	SS	44.1 ± 3.4	39.0–53.0	45.4 ± 2.9	38.5–53.0	44.7 ± 3.2	38.5–53.0	0.140
	II	43.1 ± 3.8	33.5–52.0	44.3 ± 3.0	37.0–50.0	43.6 ± 3.5	33.5–52.0	0.175
GCL volume (mm^3^)		1.09 ± 0.09	0.97–1.33	1.13 ± 0.07	0.99–1.27	1.1 ± 0.08	0.97–1.33	0.009*

**Fig. 2:**
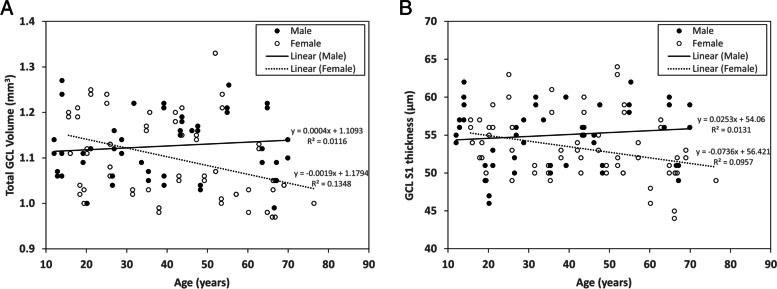
Variation of total GCL volume with age (**A**) and GCL S1 segment with age (**B**) in the entire study population, stratified by sex. Each data point represents a single eye. The regression line and equations are shown. After adjustment for axial length and sex, total GCL volume did not have an age association (*P* = 0.138); however, the association with age differed for females and males, being significant for females only (*P* = 0.003)

N = 51 eyes for males, 65 eyes for females; GCLV = total GCL volume; anatomic locations represent different sectors according to Fig. 2; *SD*  Standard deviation

Total GCL volume was significantly greater in males (1.13 ± 0.07 mm^3^) than in females (1.09 ± 0.09 mm^3^) (*P* = 0.031) after adjustment for both age and axial length. In addition, GCL thickness in C, SS, TT, NN and II was significantly higher in males than in females (Table 2) after adjustment for age and axial length (Table 3). Mixed model results for association of all sectors and regions of the GCL measurements with age, axial length, and sex are summarized in Table 3.

**Table 3:** Mixed linear model results (*P*-values) for different sectors. * *P* value < 0.05

Parameter	Age, adjusted for axial length and sex	Axial length, adjusted for age and Sex	Sex, adjusted for age and axial length
GCLC	0.603	0.195	0.000*
GCLT1	0.041*	0.508	0.052
GCLS1	0.367	0.078	0.011*
GCLN1	0.504	0.175	0.032*
GCLI1	0.296	0.334	0.027*
GCLT2	0.059	0.174	0.018*
GCLS2	0.397	0.109	0.070
GCLN2	0.64	0.26	0.250
GCLI2	0.022*	0.116	0.226
GCL inner ring	0.202	0.241	0.025*
GCL outer ring	0.125	0.6	0.660
GCLTT	0.028*	0.274	0.017*
GCLSS	0.325	0.058	0.012*
GCLNN	0.493	0.043*	0.043*
GCLII	0.081	0.171	0.039*
Total GCL Volume	0.138	0.048*	0.031*

GCL volume did not vary with age for males (Pearson *r* = 0.108, *P* = 0.45); however, for females, GCL volume declined with age (Spearman *r* = -0.404, *P* < 0.001) (Fig. 2). The GCL volume for the whole study population declined with age (*P* = 0.031) in the unadjusted data, but this association was no longer significant when adjusted for axial length and sex (*P* = 0.138) (Fig. 2).

**Fig. 3:**
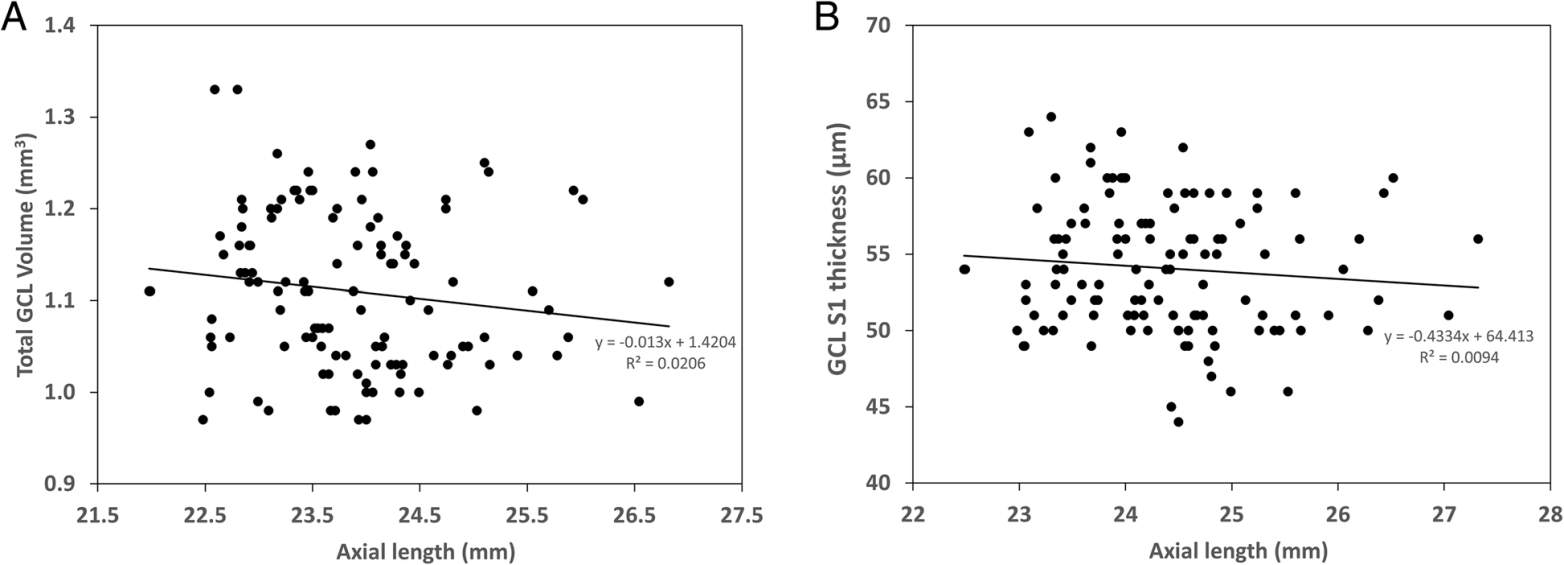
Variation of total GCL volume (**A**) and GCL S1 (**B**) with axial length in the entire study population. Each data point represents a single eye. The regression line and equation are shown. After adjustment for age and sex, reduction in total GCL volume was associated with an increase in axial length (*P* = 0.048)

The GCL volume was reduced in eyes with longer axial length, even after adjustment for age and sex (*P* = 0.048) (Fig. 3).

## Discussion

Newer OCT devices with higher precision can measure the GCL thickness in isolation without including another layer such as the IPL, and the automated OCT segmentation is correct in most of the measurements. In our study, however, we found that about 25% of the measurements needed to be manually adjusted after subjective examination of the automated segmentation by the OCT device, which has not to our knowledge, been reported in any prior study. If not corrected, automated measurements may affect the assessment of the GCL and any subsequent analyses based on this layer.

A reduction in the GCL thickness and volume with age is noted based on OCT measurements. This age effect, however, disappeared in our study when correcting for the effects of sex and axial length. Notably, two prior studies [13] and [14] reported a significant change in GCL thickness with age when adjusting for sex, but they did not measure axial length; on the other hand, in another study it was reported that GCL thickness declined with age after adjustment for axial length, but without adjusting for sex [15]. Our result emphasizes the importance of taking into consideration both axial length and sex as potential confounding factors.

In our data, thinning of the GCL layer with increasing axial length can be explained by the distribution of the nearly fixed number of ganglion cells in the retina (about 0.9–1.2 million) [17] over a larger surface area in case of a longer bulb.

Also, we found that the GCL layer was thicker in men than in women (Table 2), and that in women (but not men), GCL volume reduction with age was highly significant. Similarly, reduction in the brain volume with age has been reported [18], and studies have also shown a significant volumetric difference between men and women [19, 20]. As the ganglion cell develops from the brain, changes in thickness with both age and sex may indicate a possible association between the GCL and brain volume. The effect of sex hormones during the fetal stage or in early infancy may be implicated, as these hormones affect the neural development as well as the neuronal complexity and axonal and process formation in the brain [19, 21].

The GCL thickness difference we found in the various anatomic sectors of the inner ring indicates that the vertical sectors (superior and inferior) are the thickest followed by the nasal and then the temporal; this may reflect the migration pattern of ganglion cells at the macula [22] during development, where the cells adopt a more horizontal migration direction than vertical.

A limitation of our study is the relatively small population of healthy participants examined. Although the population size was sufficient for the statistical tests we used, ideally further studies with larger groups of healthy participants would be required to confirm our findings. However, given the range of neurological diseases reported to affect the GCL thickness and/or volume [1, 4–6, 9, 12, 23, 24], it is suggested that tests to exclude such diseases may be necessary when evaluating the GCL. Even if the possibility is very remote, despite all our measures to exclude glaucoma there may be a possibility of inadvertently enrolling a preperimetric early case of glaucoma which usually needs a follow up examination to document progression, but this was beyond our study design.

In summary, we describe the GCL thickness and volume differences and variation in different anatomic sectors of the retina according to age, sex, and axial length. Strengths of the study were our broad exclusion criteria, spatially-resolved anatomic measurements, adjustment for keratometry and spherical equivalent for each subject to improve OCT accuracy, as well as manual correction of automated OCT measurements. Based on our results, it is recommended that future studies examining GCL thickness or volume should take age, sex as well as axial length into consideration as well as the possible need for manual adjustment of automated OCT measurements.

## Data Availability

The datasets generated and/or analyzed during the current study are not publicly available due to its involvement in another study now but are available from the corresponding author on reasonable request.
